# Dynamic FDG‐PET demonstration of functional brain abnormalities

**DOI:** 10.1002/acn3.51546

**Published:** 2022-09-07

**Authors:** Mark Quigg, Bijoy Kundu

**Affiliations:** ^1^ Department of Neurology University of Virginia Charlottesville Virginia 22908 USA; ^2^ Departments of Radiology & Medical Imaging and Biomedical Engineering University of Virginia Charlottesville Virginia USA

## Abstract

Positron emission tomography with fluorine‐18 fluorodeoxyglucose (^18^F‐FDG‐PET) has been used over 3 decades to map patterns of brain glucose metabolism to evaluate normal brain function or demonstrate abnormalities of metabolism in brain disorders. Traditional PET maps patterns of absolute tracer uptake but has demonstrated shortcomings in disorders such as brain neoplasm or focal epilepsy in the ability to resolve normally from pathological tissue. In this review, we describe an alternative process of metabolic mapping, dynamic PET. This new technology quantifies the dynamics of tracer uptake and decays with the goal of improving the functional mapping of the desired metabolic activity in the target organ. We discuss technical implementation and findings of initial pilot studies in brain tumor treatment and epilepsy surgery.

## Introduction

Positron emission tomography (PET) with fluorine‐18 fluorodeoxyglucose (^18^F‐FDG) has been used over 3 decades to map patterns of brain glucose metabolism in language and cognition,^1^, ^2^ dementias,^2^ traumatic brain injury,^3^ brain tumors,^4^, ^5^ and epilepsy.^1^, ^6^, ^7^ FDG‐PET, however, has limitations in spatial resolution and in sensitivity and specificity among the various pathologies that have been historically evaluated.

In this review, we describe an alternative process of metabolic mapping, dynamic PET. This new technology quantifies the dynamics of tracer uptake and decays with the goal of improving the functional mapping of the desired metabolic activity in the target organ. We focus on functional neuroimaging with ^18^F‐FDG‐PET, the biomechanics of dynamic PET acquisition and processing, and potential applications in neurological disease with emphasis on recent work in brain neoplasm and epilepsy.

## Dynamic Versus Static Imaging

**Figure 1:**
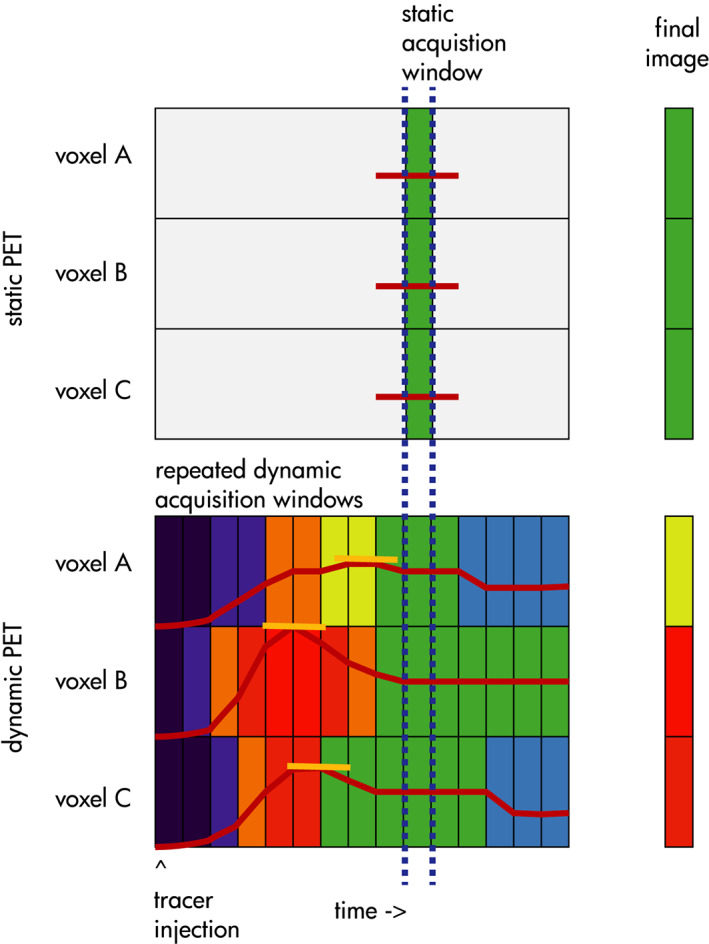
Differences between static and dynamic PET. In static PET, the absolute amount of radiation is mapped voxel‐by‐voxel during an acquisition window starting at a fixed time after completion of tracer injection. In this example, voxels A, B, and C end up with the same absolute radiation counts, measured as the standardized uptake value (SUV). In dynamic PET, tracer is captured in time windows, and analysis of kinetics (in this case, measurement of peak tracer) facilitates differentiation among voxels despite having similar absolute SUVs in the static window. [Colour figure can be viewed at wileyonlinelibrary.com]

^18^F‐FDG acts as a competitive agonist to blood glucose. It is administered intravenously, crosses the blood‐brain barrier, and mimics glucose at the cross‐membrane transport system. FDG is taken into neurons, gets phosphorylated by hexokinase, and becomes trapped within the neuron as FDG‐6‐phosphate. In static PET, the scanner maps the volumetric concentration of radioactivity within a short sampling window, typically 5 min to 2 h after injection **(**Fig. 1). Biomathematical models interpret these data to provide a quantitative, voxel‐by‐voxel map with high radiation indicating regions with the highest glucose use. The key variable returned by static clinical PET is the standardized uptake value (SUV). SUV, measured at a specific time point after FDG‐injection, provides a semi‐quantitative snapshot of glucose activity.^8^

Dynamic PET, on the other hand, measures volumetric radioactivity on a continuous basis (Fig. 1). In this paradigm, the subject is scanned at pre‐injection baseline, and the tracer is injected during active scanning. Scanning continues akin to a series of snapshots or movie frames that capture the changing concentrations of radiotracer per scanning interval. The process provides a volumetric concentration‐time profile for glucose metabolism; kinetic models measure, voxel‐by‐voxel, the rate and distribution of radioactivity uptake, decay, or tracer release. In the case of FDG‐PET, kinetic processes may be a differentiating factor because abnormal tissues may have higher or lower concentrations of hexokinase or differences in hexokinase function that, in turn, allows higher rates, lower rates, or altered rate profiles of glycolysis compared to normal tissues.

Unlike static PET, which can be mathematically described with the SUV, dynamic PET must account for the complicated kinetics of the rates of FDG uptake (Ki) and the metabolic rate of glucose uptake (MRGlu) (Table 1). These depend on the first pass effect (the liver's extraction of a proportion of the glucose analog as it passes through the venous circulation on its path to the brain) that affects the overall availability of tracer. The flux of FDG from vascular to extravascular spaces (k2) determines the availability of tracer to the neuron. Finally, the sequestration of tracer determines the amount of tracer mapped (k3). These equations required validation in animal models to provide estimates of accurate brain metabolic dynamics. These parameters may provide insight into the glucose metabolic rates and metabolic vascular heterogeneity of pathological foci and comprise key factors in dynamic image processing.

**Table 1:** Variables required to quantify metabolic mapping in dynamic PET.

Definition	Variable
First pass tracer kinetics	*K*_1_
FDG flux across vascular to extravascular spaces	*k*_2_
Sequestration of FDG in cellular spaces	*k*_3_
Empirical constant	LC
Average blood glucose	[Glu]
Rate of FDG uptake *K*_i_	=*K*_1_·*k*_3_/(*k*_2_ + *k*_3_)
Metabolic rate of glucose uptake MRGlu	=Ki·[Glu]/LC

## Acquiring Dynamic PET

**Figure 2:**
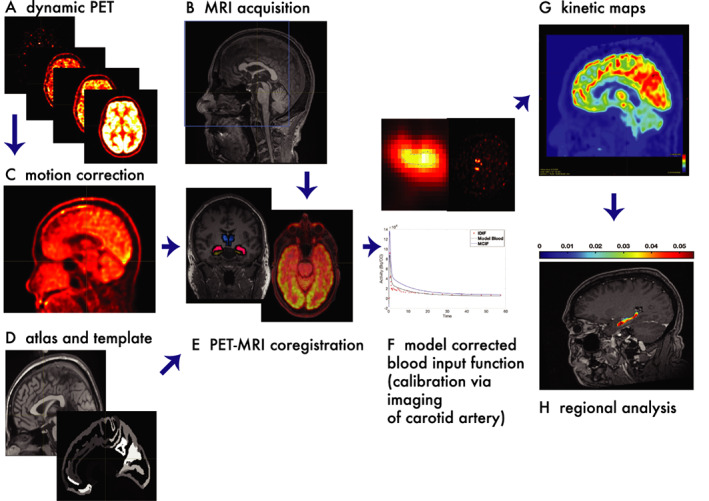
Dynamic PET (A) is preceded by a high‐resolution post‐contrast T1‐weighted (B) MPRAGE MRI (256 pixels × 256 pixels × 192 slices) using a Siemens 3 T scanner for co‐registration. PET images are obtained with a Siemens Biograph time of flight (TOF) mCT scanner. Dynamic acquisition consists of an intravenous ~10 mCi FDG tracer injection over 10 s at the start of a 60‐min scan in list‐mode format. Subsequent processing is performed with custom tools developed in Matlab (Mathworks Inc., Natick, MA) and performed using MRtrix functions.^37^ Motion correction for the 60‐min acquisition (C) is performed by averaging the first 14 frames of PET data (400 pixels × 400 pixels × 111 slices × 38‐time frames) to create a reference for a rigid body transform across subsequent frames. Motion corrected PET frames are resliced and co‐registered with T1‐weighted MRI using non‐rigid transform to generate a transformation matrix used, in turn, to generate a co‐registered dynamic PET. Next, the MRI is co‐registered with a high‐resolution T1‐weighted MRI template provided by the Montreal Neurological Institute (MNI).^38^ (D and E) using a non‐rigid transform, and a transformation matrix is generated. The total 164 regions of the Destrieux atlas^39^ defined on the same MR brain template are binned to generate regions of interest. The transformation matrix is inverted and applied to all regions of interest to move them from the MNI template into the patient MRI. The image is calibrated with the model‐corrected blood input function (MCIF) (F). Each voxel of the dynamic PET volume is then independently fed into a 4 parameter 3‐compartment model^12^ or graphical Patlak^40^ model together with the MCIF. By analyzing millions of voxels across the entire brain PET volume, a parametric kinetic map (G) is computed. On a practical note, such iterative computing is only feasible with the use of parallel computing techniques.^41^ The above regions generated in patient MR space are then applied to the computed parametric PET maps in Matlab to obtain kinetic or z‐score anatomic maps (H). [Colour figure can be viewed at wileyonlinelibrary.com]

The longer scan times and the need to calibrate concentration‐time curves require procedures that differ from static PET. The process used in our ongoing studies is described below^9^ (Fig. 2).

### Acquisition

PET is preceded by MRI for purposes of co‐registration. While the patient lays in the scanner with active scanning, intravenous FDG (dose = 10 mCi) is injected; images are collected over 60 min in list‐mode format.

### Motion correction

An unsedated patient will move over the course of 60 min. Automatic motion correction starts with an initial frame of images which is used to transform subsequent images.

### Co‐registration with MRI

The stabilized images are not only co‐registered with MRI but segmented into anatomic regions of interest with the use of published MRI atlases.

### Calibration with blood input function

Calibration of radiation counts (providing the upper and lower limits of radiation uptake) occurs with calculation of the model‐corrected blood input function (MCIF). Details are provided below.

### Kinetics mapping

kinetic mapping is applied across all voxels covering the whole brain PET volume. The computational burden is high enough to require parallel computing techniques for practical use.

## Model‐Corrected Blood Input Function

The calculation of tracer kinetics requires capturing reference values of blood tracer concentration, the blood input function. The input function describes the maximal level of radiotracer within the blood that is available for tissues to use. The ideal blood input function would be continuous intra‐arterial blood sampling over the whole period of PET acquisition obtained from a major artery. While dynamic imaging with animal models can be done with invasive intraarterial monitoring, practical human imaging requires less invasive approaches.

One method of sampling that avoids arterial access, but still requires intravenous access, is heated vein blood sampling.^10^ In this approach, modeling is used to estimate arterial FDG concentrations through repeated venous samples. In dynamic PET imaging, however, the accumulated blood sampling for an hour's duration is neither ideal nor easily tolerated.

A noninvasive approach is to acquire the blood input function through sampling of the image itself. The image‐derived blood input function, as developed by us and others^11^ uses the imaged inferior vena cava^12^ or the left ventricular blood pool^13^ as sources of maximal radioactivity in animal models.^14^ These sites serve as large, unambiguous sites that contain a readily accessible pool of blood that contains the maximal concentration of tracer for calibration of the “ceiling” of radiation.

Such large pools of blood, however, are not accessible in human brain imaging since these organs lie outside the limited field of view of PET scanners.^15^ In humans, the only “pools” available for calibration are the internal or external carotid arteries, and these are small (~6 mm) compared to the PET scanner's spatial resolution (~5 mm). Therefore, accurate calculation of the image‐derived input function can be impaired by partial volume averaging or spillover effects by the inability to separate carotid contents from surrounding tissues. Some approaches, such as reference tissue models^16^that use a reference region – the cerebellar gray matter, for example, depending on the assumption that FDG‐uptake is stable in comparison to cerebral cortex and violate the principle that no active brain region is devoid of FDG‐uptake.^17^

Practical dynamic PET imaging, therefore, requires methods to accurately record the blood input function from the image of an unsedated human subject. We have developed a model‐corrected blood input function (MCIF),^13^, ^18^ a dual output model that corrects for partial volume averaging or spillover contamination while robustly optimizing sampling. Briefly, an image‐derived blood input function is sampled and averaged from early time frames of the left internal carotid artery. These samples are applied to all the motion‐corrected 38 PET frames to generate blood time‐activity curves (*PET*_*IDIF*_) during the acquisition, mimicking the ideal continuous sampling of intraarrterial monitoring. A model IDIF that also corrects for spillover contamination consists of:

(1) $${\text{Model}}_{\text{IDIF},i}=\frac{\int_{t_{b}^{i}}^{t_{e}^{i}}\left[S_{\mathrm{Tb}}C_{T}\left(t\right)+r_{b}C_{a}\left(t\right)\right]\mathrm{dt}}{t_{e}^{i}-t_{b}^{i}},$$

in which *S*_*Tb*_ = spillover contamination from the tissue to the blood at late time points, *r*_*b*_ = blood recovery coefficient, *t*_*b*_ and *t*_*e*_ = beginning and end of a time frame. *C*_*T*_ *(t),* the model tissue, was obtained by solving FDG‐transport differential equations from blood to tissue spaces as described. *C*_*a*_ *(t)* is 7‐parameter model blood for FDG‐transport as described. The above model IDIF is then optimized using the following objective functions:

(2) $$O_{1}\left(p\right)=\sum_{i=1}^{n}{\left({\text{Model}}_{\text{IDIF},i}-{\mathrm{PET}}_{\text{IDIF},i}\right)}^{2}$$

(3) $$O_{2}\left(p\right)={\left({\text{ModelPeak}}_{\text{IDIF}}-{\text{PETPeak}}_{\text{IDIF}}\right)}^{2}$$

(4) $$O\left(p\right)=O_{1}\left(p\right)+O_{2}\left(p\right).$$

ModelPeak is computed from the model equations for the IDIF (Model_IDIF_) (Equation 1). PETPeak values are derived from the dynamic PET blood images for each patient. Optimization of *O(p)* used non‐linear regression analysis yielding the estimate of MCIF. Computation of MCIF is a semi‐automated process with a priori determination of the lower and upper bounds and the initial guess values of the parameters, an approach recently validated with arterial blood sampling in rodent total body dynamic PET imaging (see details below).^14^

## Technical Studies

Animal work has validated processes important in subsequent human studies. Computation of a model blood input function was validated against “gold‐standard” arterial blood sampling of the left ventricular blood pool in rodents as described.^14^ Rodent cerebral and cardiac FDG‐PET studies using normal rats have validated the underlying technical components of dynamic PET. These technical steps include (1) computation of the blood input function and (2) rate of tissue FDG uptake.^12^, ^14^

Animal studies have also established high intrasubject reliability, ensuring that resting‐state dynamic FDG‐PET acquisitions do not feature idiosyncratic changes in hyper‐ or hypometabolism. Computed myocardial FDG uptake rates of control rat heart repeated over the entire life cycle from 1 month to 18 months of age^19^, ^20^ (measurements at 1, 2, 3, 5, 9, 12, and 18 months) was 0.0189 ± 0.006 min^−1^. The overall coefficient of variance of 0.3 indicates high intrasubject repeatability in the estimate of the kinetic parameter.

Some early human studies performed in our lab established practical parameters for subsequent use. For example, we determined that a 1 h scan time was a reasonable duration of acquisition to both collect sufficient serial data while recognizing the ability of patients to tolerate an unsedated study. We have no experience in obtaining studies with the aid of conscious sedation, but certainly, if to be used in those with cognitive impairment or in pediatric cases, conscious sedation may be required. Furthermore, in our cases with epilepsy, the possibility of seizure rises with the duration of scan so we perform those studies with simultaneous EEG monitoring with the use of scanner‐compatible electrodes.

## Dynamic Brain PET in Human Brain Cancer

Glioblastoma multiforme (GBM) is a malignant, infiltrating, primary brain tumor that has an annual incidence of 3.19 cases per 100,000 person‐years and poor prognosis with a 5‐year survival rate of 4%–5%.^21^ Static FDG‐PET has been evaluated as a test to differentiate among infiltrating tumors from inflamed posttreatment tissues or normal brain. Unfortunately, in the case of GBM, SUV calculated from static FDG‐PET does not reliably differentiate tumor from post‐therapy changes.^22^ Although actively growing tumors avidly consume glucose, other pathological processes such as inflammation or infection also feature high glucose uptake. In addition, body weight and blood glucose level can complicate the interpretation of SUV.^23^ Attempts to address this shortcoming include the use of ligands other than FDG. For example, since l‐amino acid transporters are overexpressed in many gliomas,^24^ pilot studies or larger series have evaluated amino acid ligand including 3,4‐dihydroxy‐6‐[^18^F]fluoro‐l‐phenylalanine (^18^F‐FDOPA),^25^ 11C‐methionine (11C‐MET), and ^18^F‐fluoroethyl‐l‐tyrosine (^18^F‐FET).^26^ Although most studies indicate that the studied radiotracers offer some benefits over FDG, none of them appear to have a clear advantage over the others.^27^, ^28^, ^29^, ^30^

In this setting, dynamic PET remains an important option to consider. For example, recent work by Wardak et al^31^ used dynamic PET and tracers of cell proliferation or l‐amino acid analogs to statistically model survival in patients with recurrent malignant gliomas. They found that kinetic parameters obtained early after the start of treatment (absolute values and their associated changes) provided sufficient information to predict the outcome with reasonable confidence using regression techniques.

**Figure 3:**
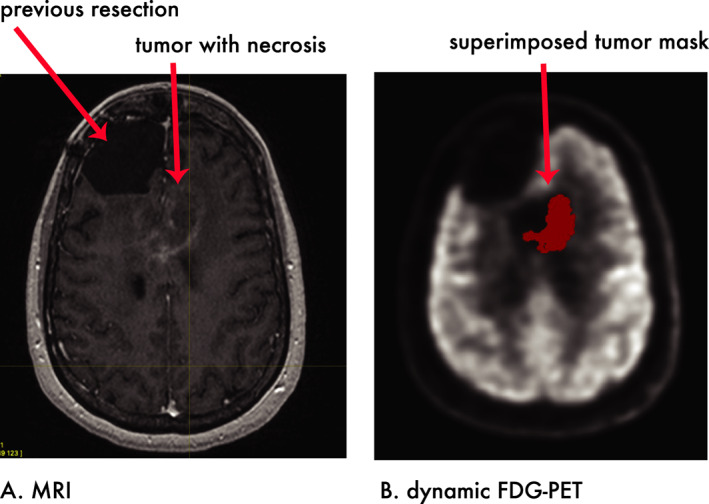
Dynamic FDG‐PET in brain tumor treatment. (A) MRI provides anatomic structure of the tumor which, (B) when co‐registered upon dynamic FDG‐PET with the locations and shapes of tumors, provides local measures of glucose kinetics within each tumor. Preliminary work established that tumor‐site‐associated glucose kinetics can help create statistical models that better differentiate tumor progression from radiation‐induced inflammation.

Preliminary work from our laboratory has explored the problem of differentiating tumor progression from treatment‐related necrosis with the use of predictive algorithms based on dynamic FDG‐PET.^32^ In a pilot study to develop a predictive model, dynamic FDG‐PET was performed on 25 patients with GBM who had undergone a range of treatments including surgical resection followed by chemoradiotherapy. Patients underwent dynamic FDG‐PET imaging as outlined earlier. T1‐weighted MRI not only supplied information for co‐registration but also provided three‐dimensional maps (“masks”) of tumor location and volumes. MRI‐tumor masks were co‐registered to parametric dynamic PET maps to generate average kinetic rate constants (K_1_‐k_3_ and K_i_) and total blood volume (TBV) for each tumor for each patient (Fig. 3).

To provide objective distinctions between tumor and normal tissue, we created a statistical model with the dependent outcome of lesion status, coded as tumor progression or tumor necrosis, for any given patient based on gold standards of expert interpretation of MR imaging obtained in follow up (*n* = 25) or, when available, the combination of follow‐up imaging with surgical pathology (*n* = 7). The lesion‐related kinetic rate constants provided the independent variables of predictive input values. After creating the model with total of 34 lesions (tumors = 23, necrosis = 11) across 25 patients, the resulting model predicted lesion type from a test set. The model accurately predicted 95% of tumors and 75% of necrotic lesions. This pilot study suggests dynamic FDG‐PET imaging could improve the diagnostic accuracy of brain tumor treatment.

## Dynamic FDG‐PET in Localization of Focal Epilepsy

Thirty to 40% of patients with epilepsy continue to have seizures that are not controlled by medications.^33^ Best‐practice guidelines recommend that patients with medically intractable epilepsy undergo evaluation for epilepsy surgery with the goal of identifying a seizure focus.^34^ Identifying the seizure focus is straightforward when a lesion is identified on MRI and other information is concordant with that finding. However, many patients with seizures identified by focal changes on the EEG do not have a lesion on MRI. In that subset of “lesionless focal epilepsy”, subsequent surgery, usually requiring invasive monitoring techniques, has a worse outcome compared to those with visible focal lesions present on imaging.^35^ Thus, enhancing the ability to locate seizure foci in focal epilepsy patients is important to identify surgical targets to improve outcomes.

FDG‐PET forms an important part of the noninvasive stage of presurgical localization because it measures neuronal metabolism rather than anatomy as is done by MRI, or electrical activity as is done by EEG. Metabolically hypoactive regions revealed by decreased glucose uptake correspond to seizure foci on routine interictal static PET obtained between seizures. But, standard, static PET has limitations of a relatively low rate of accurate identification of seizure foci and relatively low resolution.

To date, only two studies have piloted the use of dynamic FDG‐PET in patients with medically intractable focal epilepsy. One recruited 17 patients being considered for epilepsy surgery along with 8 control subjects. All had standard, static FDG‐PET images extracted from dynamic FDG‐PET; all patients had identifiable regions of focal hypometabolism on static FDG‐PET.^36^ These patients all underwent epilepsy surgery and were found to be seizure‐free on follow‐up at least 1 year after surgery. Retrospectively, the pre‐operative dynamic FDG‐PET images were visually reviewed by experts blinded to clinical findings. The authors determined that regions of hypometabolism were similar to those seen on static PET in patients with epilepsy and that no “false positive” regions of hypometabolism were seen in controls. This study, however, did not explicitly evaluate possible advantages in the use of dynamic FDG‐PET.

**Figure 4:**
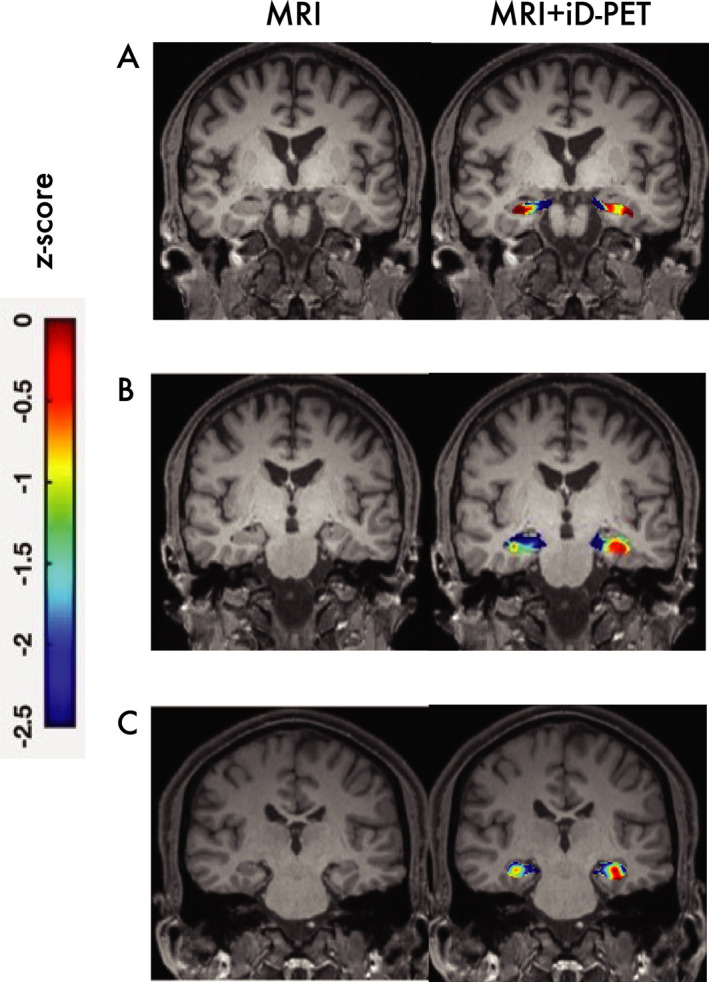
T1‐weighted MRI (left side images) and co‐registered dynamic FDG‐PET maps of hypometabolism by z score (right images) from epilepsy patients whose standard, static FDG‐PET images were normal. (A) This patient underwent a previous right anterior frontal lobotomy with no improvement in seizure frequency. iD‐PET demonstrated relative right hippocampal hypometabolism; subsequent intracranial monitoring confirmed seizure onset in the right hippocampus. (B) This patient had evidence of bilateral mesial temporal lobe epilepsy. iD‐PET disclosed worse right hippocampal hypometabolism; this patient declined further surgical intervention. (C) Patient with normal MRI and poorly localizing seizures with worse right hippocampal hypometabolism. This patient underwent thermal ablation of the right hippocampus and has long‐term seizure remission.^9^

We recently completed a pilot study that recruited epilepsy surgery candidates who had normal, static FDG‐PET to determine if dynamic FDG‐PET would be more sensitive in the identification of clinically appropriate regions of focal glucose hypometabolism.^9^ In our study, 9 patients underwent dynamic FDG‐PET from which a static study was extracted. One patient was excluded because of a seizure during acquisition. Technical problems prevented another from obtaining MRI for co‐registration. In the remaining 7 patients, focal hypometabolism (quantitatively labeled as having at least a 2 standard deviation decrease in kinetic markers in side‐side comparisons) was identified in all **(**Fig. 4). Note that standard, static PET used in evaluation for epilepsy surgery was normal; therefore, 100% of subjects in this small study had focal findings on dynamic FDG‐PET that were not present on static PET.

Patients fell into three groups according to relationships between proposed localization (via Epilepsy Surgery Committee evaluation) and dynamic FDG‐PET. Four had unilateral mesial temporal/hippocampal regions of hypometabolism on dynamic FDG‐PET concordant with an anticipated surgical target; one underwent laser interstitial thermal therapy of the ipsilateral hippocampus at the site of dynamic FDG‐PET hypometabolism and was seizure‐free for 30 months at last follow‐up. These patients represent the potential of improved sensitivity of determination of surgical targets.

A patient who had previous unsuccessful right frontal lobectomy had right mesial temporal hypometabolism on dynamic FDG‐PET. Subsequent intracranial monitoring disclosed multifocal seizure foci. This patient represents the potential ability of dynamic PET to provide a prognosis against further surgical intervention.

Finally, three patients had dFDG‐PET findings indicating unilateral mesial temporal hypometabolism in the context of bilateral mesial foci proposed by the Epilepsy Surgery Committee review. Each declined intracranial monitoring. These two patients represent the potential of dynamic PET to guide invasive evaluations to bring more patients to future surgery.

Overall, the pilot study suggests that dynamic FDG‐PET may indicate focal regions of hypometabolism in epilepsy surgery subjects whose standard static FDG‐PET was unhelpful.

From a technical standpoint, the second study offers several innovations that could improve subsequent work. Our use of a semi‐automated, objective procedure of model‐corrected blood input function offers practical and theoretical advantages over manually selected initial images of the internal carotid artery. This innovation turns what in the past was an operator‐dependent and manually‐calculated process into a rigorous, semi‐automated method that has not only informed our preliminary work but has been rigorously validated in a prior animal‐model work with arterial blood sampling.^13^, ^14^ Additionally, this pilot study indicated no significant operator variability in the computed FDG uptake rates with a robust coefficient of variance (0.2) with voxel‐by‐voxel changes of less than 3%.

## Conclusion

In summary, this review has provided an overview of the biological and technical underpinnings on how dynamic PET can better capture functional differences in normal versus pathological brain regions. Recent technical innovations may facilitate easier and more widespread use of dynamic PET. Current work has centered on brain neoplasm and focal epilepsy; future work may yield promising results in degenerative disease, such as dementia, traumatic brain injury, or neurovascular disorders.

## Conflicts of Interest

The authors report no conflicts of interest.

## Author Contributions

Dr Quigg conceptualized and undertook the human research, wrote and edited the manuscript.

Dr Kundu developed and undertook engineering, human, and animal research, and wrote and edited the manuscript.
